# Reappraisal of soft tissue myoepithelial tumors by DNA methylation profiling reveals an epigenetically distinct group of mostly fusion-driven neoplasms

**DOI:** 10.1007/s00428-024-03977-4

**Published:** 2024-12-05

**Authors:** Faizan Malik, Selene C. Koo, Nasir Ud Din, Quynh T. Tran, Oscar Lopez-Nunez, Sabina Barresi, Silvia Vallese, Giuseppe Milano, Evelina Miele, Michael R. Clay, Rita Alaggio, Brent A. Orr

**Affiliations:** 1 Department of Pathology, MS 250, St. Jude Children’s Research Hospital, 262 Danny Thomas Pl, Memphis, TN 38105 USA; 2 Department of Pathology and Laboratory Medicine, Aga Khan University Hospital, Karachi, Pakistan; 3 Department of Pathology, Cincinnati Children’s Hospital Medical Center, Cincinnati, OH USA; 4 Pathology Unit, Ospedale Pediatrico Bambino Gesù, IRCCS, Rome, Italy; 5 Oncology Unit, Ospedale Pediatrico Bambino Gesù, IRCCS, Rome, Italy; 6 Department of Pathology, University of Colorado, Anschutz Medical Campus, Aurora, CO USA

**Keywords:** Myoepithelioma, Myoepithelial tumor, DNA methylation, EWSR1, KLF15, PBX3

## Abstract

Soft tissue myoepithelial tumors (METs) are diagnostically challenging tumors that require careful histologic and immunohistochemical characterization for accurate classification. Nearly half of METs show recurrent *EWSR1* or *FUS* gene rearrangements with a diverse set of fusion partners. The diversity of fusion partners and lack of known driver abnormalities in many cases raises the question of whether METs represent a uniformly distinct tumor entity. To address this question, we performed careful histopathologic and molecular analysis, including DNA methylation profiling (DNA-MP) and fusion testing, on a cohort of 30 institutionally diagnosed METs from 29 patients. On histologic and immunophenotypic evaluation, 22 of 30 tumors diagnosed as MET fulfilled strict histologic and immunophenotypic criteria. Among those failing to meet criteria, most were reclassified as another tumor entity by DNA-MP. Seven tumors meeting criteria grouped with another sarcoma reference type by DNA-MP, with confirmation of the characteristic driver abnormality of that tumor in selected cases. The remaining tumors histologically “consistent” with METs (*n* = 15) formed a distinct epigenetic cluster, independent of other reference entities. Recurrent gene fusions were identified in 11 of 15 tumors in this epigenetically distinct group, including *EWSR1*::*KLF15* (*n* = 4), *EWSR1*::*PBX3* (*n* = 2), and *EWSR1*::*POU5F1* (*n* = 1) rearrangements. Clinicopathologic correlation suggests that *EWSR1*::*KLF15* tumors are enriched in pediatric patients with aggressive histology. Our work shows that at least a subset of METs falls within an epigenetically distinct but heterogenous group. Furthermore, DNA-MP provides a useful adjunct to other molecular testing to help distinguish METs from histologic mimics.

**Supplementary Information:**

The online version contains supplementary material available at 10.1007/s00428-024-03977-4.

## Introduction

Myoepithelial tumors (METs) of soft tissue are rare and morphologically diverse neoplasms [1]. Accurate classification requires demonstration of the “myoepithelial immunophenotype,” defined by co-expression of epithelial antigens [epithelial membrane antigen (EMA) or cytokeratins (CK)] and S100, SOX10, or glial fibrillary acidic protein (GFAP), in the correct morphologic context [2]. Although gene fusions have been described for METs, including EWS RNA binding protein 1 (*EWSR1*) and FUS RNA binding protein (*FUS*) fusions, these are found in less than 50% of cases [3]. Even in fusion-positive METs, the reported partners are heterogenous and include POU class 5 homeobox 1 (*POU5F1*), PBX homeobox 1 (*PBX1*), *PBX3*, zinc finger protein 444 (*ZNF444*), KLF transcription factor 15 (*KLF15*), *KLF17*, or activating transcription factor 1 (*ATF1*) [3–8]. For fusion-negative METs, recurrent driver abnormalities remain undefined. Recent studies have suggested that, in some instances, fusion-negative METs may represent alternative tumor types [9]. These observations have led to some uncertainty around whether METs represent a distinct entity, causing diagnostic uncertainty in cases with suboptimal antigenic expression, or when molecular testing is inconclusive or unavailable.

Genome-wide DNA methylation profiling (DNA-MP) is a powerful tool that has been used to refine the molecular classification of brain and soft tissue tumors [10, 11]. A tumor’s DNA methylation signature is thought to represent a combination of the cell of origin and the specific driver abnormality. The most common platform used to interrogate genome-wide DNA methylation signatures is the Illumina Infinium BeadChip Array. These arrays have been used to characterize molecular subgroups in morphologically homogeneous tumor entities, to distinguish between tumors of different histotypes with overlapping genetics, and to identify prognostic subgroups within tumor types [10, 12–14]. Tumor DNA methylation signatures have been used for clinical diagnostics by training supervised machine learning models on reference datasets of both brain tumors and sarcomas to help predict the molecular class of clinical tumor samples [10, 11].

The morphologic and molecular heterogeneity of METs indicates a need for better methods of investigating and classifying these tumors. In this study, we constructed an international cohort of institutionally diagnosed soft tissue METs. We evaluated the clinicopathologic and molecular features of this cohort, including the epigenetic profiles and gene fusion status, to determine the molecular distinction between METs and other soft tissue tumors [11].

## Methods

The study was approved by the institutional review board at each participating institution (St. Jude Children’s Research Hospital, United States; Ospedale Pediatrico Bambino Gesù, Italy; and Aga Khan University Hospital, Pakistan). We collected a cohort of institutionally diagnosed METs (benign and malignant) between 2010 and 2022. The cases were diagnosed by experienced pathologists with sarcoma diagnostic expertise (M.R.C., N.U.D., R.A.) after careful characterization of clinical; histological; immunohistochemical; and, where available, molecular findings. The available material was re-reviewed by two pathologists (F.M. and S.C.K.) to summarize histologic and IHC features. Tumors were categorized as histologically “consistent” when they showed the WHO essential diagnostic criteria, i.e., (1) compatible histology (trabecular, reticular, nested, and/or solid growth of variably spindled or epithelioid cells with a frequent myxoid or hyalinized stroma) and (2) immunohistochemical expression of epithelial markers (CKAE1/3 and/or EMA) with at least one additional myoepithelial marker (S100, SOX10, or GFAP) [1, 2]; positivity for these markers was interpreted as focal (moderate to strong staining in 10–50% of tumor cells) or diffuse (moderate to strong staining in more than 50% of tumor cells) and assessed to disregard any spurious or artifactual staining. Tumors not meeting these criteria were placed in the histologically “questionable” group. Although a broad panel of immunostains was performed in most cases at diagnosis, we included only the pertinent stains in our review. Tumors with and without molecular profiling were included for two reasons. First, more than half of METs are fusion-negative, and second, a subset of included cases was diagnosed either prior to routine use of diagnostic molecular pathology assays or in resource-limited settings without access to these assays. Clinical and follow-up information was retrieved from clinical records, where available. To assess the potential epigenetic and molecular relationship between tumors with “consistent” and “questionable” histology, all tumors were included in the analysis of this study.

### DNA methylation profiling, unsupervised analysis, molecular fusion analysis, and immunohistochemistry

Methylation analysis was performed using the Illumina Infinium Human MethylationEPIC BeadChip (850 K) on 250–500 ng of DNA extracted from formalin-fixed paraffin-embedded (FFPE) tissue. Unsupervised analysis of methylation data was performed by hierarchical clustering and t-distributed stochastic neighbor embedding (t-SNE). Hierarchical clustering was performed and visualized using the R *circlize* (v 0.4.16) package. This approach was used for the combined analysis of sarcoma reference samples GSE140686 [11] and the 30 MET cases. Copy number profiles were generated from genome-wide DNA methylation data using the *conumee* package (v1.36.0) in R [15] as previously described [12], using the threshold values of 0.18 for copy number gain and − 0.2 for copy number loss (see Supplementary file 1 for details).

RNA sequencing (RNA-seq) was attempted on all tumors with sufficient material (see Supplementary file 1 for details). In one case, fluorescence in situ hybridization (FISH) was performed on representative FFPE tumor sections by using a commercially available capicua transcriptional repressor (*CIC*) (19q13.2) dual-color [3′ telomeric (SpecOrange)/5′ centromeric (SpecGreen)], break-apart probe (Empire Genomics; catalog number CIC-BA). All molecular results were reviewed by an experienced board-certified molecular pathologist (S.C.K.).

Additional immunohistochemical stains were performed for case re-evaluation using clinically validated protocols (see Supplementary file 1 for details).

## Results

A total of 30 institutionally diagnosed MET cases from 29 patients were collected (Table 1 and Supplementary file 2/ Supplementary Table 1). These included biopsies (15 of 30 cases) and excisions (15 of 30 cases) of 21 (70%) malignant and 9 (30%) benign tumors. Cases 1 and 2 were from the same patient’s primary pre-treatment tumor and post-therapy recurrence.

**Table 1:** Unsupervised DNA methylation analysis of myoepithelial tumors and other tumors with myoepithelial features

Case #	Pertinent IHC results								Histologic category	Hierarchical cluster	t-SNE group	RNA-seq results
	CKAE1/3	EMA	S100	SOX10	GFAP	P63	SMA	Other				
Case 1	+	+	+	N/A	+ (f)	-	+	SMARCB1 retained	Histologic Consistent	“MET” group	Small group	*EWSR1*::*KLF15*
Case 2	+	+	+	N/A	+ (f)	-	+ (f)	SMARCB1 retained	Histologic Consistent	“MET” group	Small group	*EWSR1*::*KLF15*
Case 3	+	+	+	+	-	-	-	N/A	Histologic Consistent	“MET” group	Small group	*EWSR1*::*KLF15*
Case 4	+	+	+	+	-	-	+	N/A	Histologic Consistent	“MET” group	Small group	*EWSR1*::*KLF15*
Case 5	-	+	+	-	+ (f)	+ (f)	+	N/A	Histologic Consistent	“MET” group	Individual	*EWSR1*::*PBX3*
Case 6	+	+	+	-	-	-	+	N/A	Histologic Consistent	“MET” group	Individual	*EWSR1*::*PBX3*
Case 7	-	+	+	N/A	-	-	-	SMARCB1 retained	Histologic Consistent	“MET” group	Individual	*EWSR1*::*POU5F1*
Case 8	+	+	+	+	-	-	+ (f)	N/A	Histologic Consistent	“MET” group	Individual, spatially close to CSA-A	*IRF2BP2*::*CDX1*
Case 9	+	N/A	+	-	-	-	-	N/A	Histologic Consistent	“MET” group	Individual, spatially close to CSA-A	*IRF2BP2*::*CDX1*
Case 10	+	+	+ (f)	-	-	-	-	CD99 − /MUC4 − /NKX2.2 +	Histologic Consistent	“MET” group	Individual, spatially close to SEF	*EWSR1*::*NFATC2*
Case 11	+	+	+ (f)	+	+	-	+ (f)	N/A	Histologic Consistent	“MET” group	Individual	*EWSR1* rearranged by FISH; RNA-seq N/A
Case 12	+	+	+	+	+	-	-	N/A	Histologic Consistent	“MET” group	Individual	Negative for fusions
Case 13	+	-	+	+	-	+	-	Melan-A − HMB-45 − /MiTF −	Histologic Consistent	“MET” group	Individual, spatially close to cutaneous melanoma	Negative for fusions
Case 14	+	+	+	+	+	-	+	SMARCB1 and SMARCA4 retained	Histologic Consistent	“MET” group	Individual	Negative for fusions; *EWSR1* intact by FISH**
Case 15	+	+	-	+	-	+	+	N/A	Histologic Consistent	“MET” group	Individual	RNA-seq N/A
Case 16^b^	-	+	+	-	-	-	-	CD34 − /ER − / PR − /SMARCB1 lost	Histologic Consistent	ES	ES	Negative
Case 17^b^	-	+	+	+	-	+	+	SMARCB1 lost	Histologic Consistent	ES	ES	Negative
Case 18^b^	+	+	+ (f)	-	-	-	-	SMARCB1 lost	Histologic Consistent	MRT	MRT	Negative
Case 19^b^	-	+	+	N/A	-	-	+	N/P	Histologic Consistent	EMCS	Independent	Negative
Case 20^b^	+	+	+ (f)	+	+	-	+	N/A	Histologic Consistent	OFMT	HG-ESS	N/A
Case 21^a^	-	+	+	-	-	-	+ (f)	N/A	Histologic Consistent	EMCS	EMCS	*TAF15*::*NR4A3*
Case 22^a^	+	+	+ (f)	-	-	-	-	N/A	Histologic Consistent	Synovial sarcoma	Synovial Sarcoma	*SS18*::*SSX2*
Case 23^a^	+	+	-	-	-	-	+	Desmin + , mosaic SMARCB1 lost	Histologic Questionable	OFMT	OFMT	*PHF1*::*TFE3*
Case 24^a^	-	+	-	-	-	-	+	CD99 + /WT-1 + /DUX4 +	Histologic Questionable	SBRCT (CIC)	SBRCT (CIC)	N/A; *CIC* FISH failed (poor probe hybridization)
Case 25^b^	+	+	-	N/A	-	-	-	N/A	Histologic Questionable	ES	Independent	Negative
Case 26^b^	-	+	-	N/A	-	-	-	SMARCB1 retained	Histologic Questionable	MRT	MRT	N/A
Case 27^b^	+	+	-	N/A	-	+	-	SMARCB1 and SMARCA4 retained	Histologic Questionable	MRT	MRT	Negative
Case 28^b^	-	-	+	-	-	-	+	N/A	Histologic Questionable	OFMT	OFMT	N/A
Case 29^b^	+ (f)	-	-	-	-	+ (f)	+	H3K27me3 failed (repeated)	Histologic Questionable	MPNST	MPNST	Negative
Case 30^b^	-	+	+	-	-	+	+	SMARCB1 lost	Histologic Questionable	EMCS	Independent	Negative

*CSA-A* chondrosarcoma (subtype A), *EMCS* extraskeletal mesenchymal chondrosarcoma, *ES* epithelioid sarcoma, (*f*) focal, *FISH* fluorescence in situ hybridization, *IHC* immunohistochemistry, *MET* myoepithelial tumor, *MPNST* malignant peripheral nerve sheath tumor, *MRT* malignant rhabdoid tumor, *Neg* negative, *N/A* not available or not applicable, *OFMT* ossifying fibromyxoid tumor, *Pos* positive, *SEF* sclerosing epithelioid fibrosarcoma, *SRBCT* small round blue cell tumor, *URCS* undifferentiated round cell sarcoma

^a^DNA methylation diagnosis confirmed by RNA seq or IHC

^b^DNA methylation diagnosis unresolved between MET and predicted tumor because of insufficient histologic and IHC features

First, we performed morphologic and immunophenotypic review to place tumors into a histologically “consistent” or histologically “questionable” category. On strict histopathologic review, 22 of 30 cases showed morphologic and IHC features consistent with METs (histologically consistent group), while 8 tumors failed to either meet histologic criteria or show a specific myoepithelial immunophenotype (histologically questionable group).

Tumors in the consistent group commonly showed epithelioid and spindled tumor cells within varying amounts of hyaline and myxoid matrix. Cartilage was seen in rare cases. Most tumors showed a nested and reticular architecture, with few showing solid aggregates. By IHC, the tumor cells consistently expressed EMA (20 of 21) and CKAE1/3 (16 of 22), in combination with S100 (21 of 22) or SOX10 (9 of 19). GFAP was positive in 7 of 22 cases, p63 in 4 of 22 cases, and SMA in 12 of 24 cases. EMA was positive in all 6 CK negative cases. Molecular information was available at the time of case accrual for 5 cases (cases 1–3, 11, and 14). All 22 cases were included in the final DNA-MP analysis.

The remaining eight tumors in the questionable group showed some features overlapping with METs, including epithelioid (three cases) and spindled (two cases) tumor cells with myxohyaline stroma. Immunohistochemically, six of eight tumors showed epithelial marker expression, two of eight showed S100 positivity, GFAP was negative in all eight cases, and SOX10 was negative in all five tumors tested. Overall, in many of the institutionally diagnosed METs in the questionable group, the diagnoses were based on morphologic features and co-expression of epithelial markers with other non-specific antigens seen in METs, including SMA (positive in four of eight) and p63 (positive in three of eight). One case (case 30) showed a MET immunoprofile but had an incompatible morphology. All eight cases were included in the final DNA-MP analysis for investigation of their potential relationship to histologically consistent METs.

### DNA methylation profiling

**Fig. 1:**
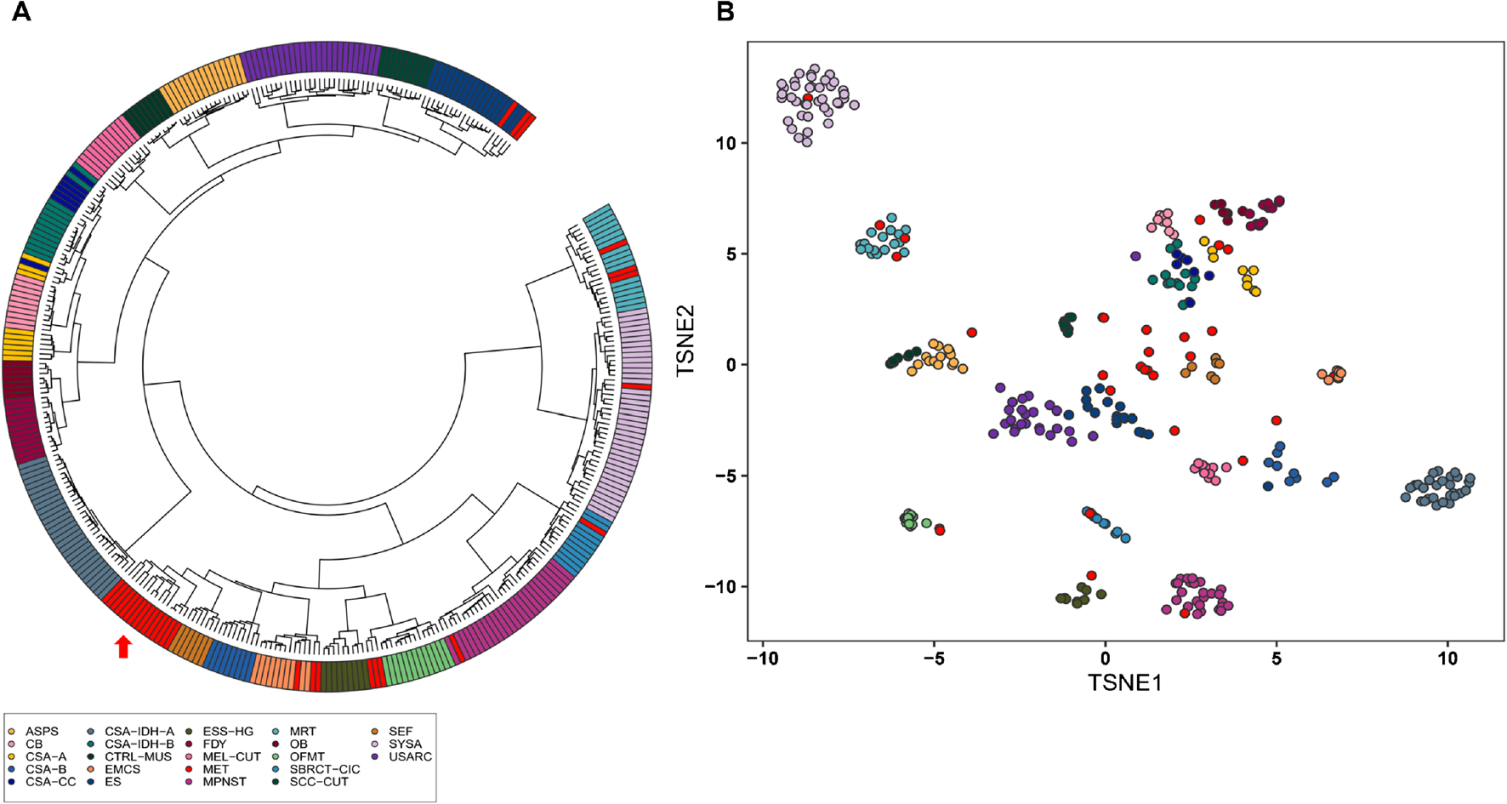
Unsupervised analysis of institutionally classified myoepithelial tumor (MET) samples. **A** Hierarchical clustering of METs with reference entities showed an epigenetically distinct subgroup that included 15 cases (red arrow). The remaining tumors in the cohort (*n* = 15) clustered with other reference tumor types. **B** The t-SNE dimensionality reduction analysis showed that most cases were widely dispersed, but a small subset formed a tighter grouping (center of plot). The subset of cases that showed hierarchical clustering with other reference tumor types similarly grouped with those entities by t-SNE. Color key includes the reference tumor entities.^11^ included in the unsupervised analysis; red dots and bars denote the MET study cohort. For annotation of fusion-positive cases, please refer to Supplementary file 3; Fig. S1. (ASPS, alveolar soft part sarcoma; CB, chondroblastoma; CSA, chondrosarcoma; CSA-CC, clear cell chondrosarcoma; CSA-IDH, chondrosarcoma (IDH mutant); CTRL-MUS, skeletal muscle control; EMCS, extraskeletal myxoid chondrosarcoma; ES, epithelioid sarcoma; ESS-HG, high-grade endometrial stromal sarcoma; FDY, fibrous dysplasia; MEL-CUT, cutaneous melanoma; MPNST, malignant peripheral nerve sheath tumor; MRT, malignant rhabdoid tumor; OB, osteoblastoma; OFMT, ossifying fibromyxoid tumor; SBRCT-CIC, capicua transcriptional repressor (*CIC*)-rearranged small blue round cell tumor; SCC-CUT, cutaneous squamous cell carcinoma; SEF, sclerosing epithelioid fibrosarcoma; SYSA, synovial sarcoma; USARC, undifferentiated sarcoma)

Next, to evaluate the epigenetic relationship between tumors diagnosed as MET and other soft tissue tumors, we performed unsupervised hierarchical cluster analysis and t-SNE of our MET cohort in comparison with reference soft tissue tumors [11]. Tumors in both histologic categories (consistent and questionable) were included in this analysis to investigate the relationship of morphology, immunotype, and molecular features. Hierarchical clustering analysis showed that 15 of 30 tumors formed epigenetically distinct group independent of the reference tumors (Fig. 1A), while the remaining 15 cases grouped with other known reference tumors, including malignant rhabdoid tumor (MRT), epithelioid sarcoma (ES), and extraskeletal myxoid chondrosarcoma (EMCS) (Fig. 1A; Table 1; Supplementary file 3; Fig. S1). The t-SNE projections largely recapitulated the hierarchical clustering. Notably, the tumors corresponding with the epigenetically distinct group by hierarchical analysis showed loose projections into the unsupervised t-SNE space. The remaining 15 cases grouped similarly to the known reference tumors as observed in the hierarchical cluster analysis (Fig. 1B; Table 1; Supplementary file 3; Fig. S1). Upon comparing the histologic categories to the unsupervised epigenetic analysis, we observed that all eight tumors in the questionable category grouped with known reference tumors. In contrast, 15 of 22 tumors that met strict histomorphologic MET criteria were present in the epigenetically distinct group.

The subset of tumors reclassified by DNA-MP (*n* = 15) as another entity included 7 tumors that met strict histomorphologic MET criteria (consistent MET) and 8 that did not (questionable MET). To validate the clustering, we performed confirmatory testing on tumors with available material. RNA-seq identified fusions in 3 tumors that confirmed the DNA-MP-predicted diagnoses. These included EMCS and synovial sarcoma (both consistent METs) and ossifying fibromyxoid tumor [(OFMT); questionable MET], respectively. For the remaining cases, tumor type–defining molecular abnormalities either do not exist (7 of 12 cases) or could not be identified in our cohort (5 of 12 cases).

The molecularly confirmed reclassified tumors showed histologic and IHC features serving as pitfalls for misclassification (Supplementary file 3; Fig. S2-S4). Molecular profiling for these tumors was unavailable at diagnosis. Both resolved cases of EMCS and synovial sarcoma showed epithelial marker expression and “myoepithelioma-like” features [16, 17]. Taken together, a subset of tumors institutionally diagnosed as MET can be reclassified as another soft tissue tumor type. Although using strict histomorphologic criteria reduced the misclassification rate (32%), we still observed cases that met histologic criteria for MET but were molecularly reclassified.

### Clinicopathologic and molecular features of epigenetically distinct group of myoepithelial tumors

To further characterize the subset of tumors in the epigenetically distinct group (*n* = 15), we performed molecular fusion analysis.

**Fig. 2:**
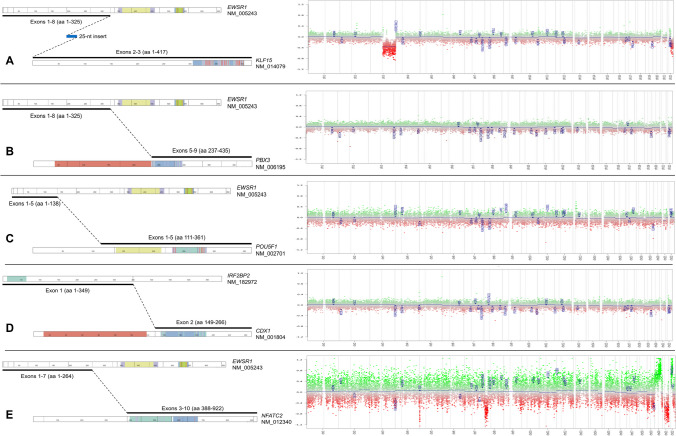
Fusion transcripts (left) and copy number profiles (right) identified in the epigenetically distinct myoepithelial tumor cohort. Tumors with recurrent *EWSR1*::*KLF15* fusions (**A**) displayed identical copy number alterations, with whole chromosome loss of 3q and partial loss of 22q. Tumors with *EWSR1*::*PBX3* (**B**), *EWSR1*::*POU5F1* (**C**), or *IRF2BP2*::*CDX1* (**D**) gene rearrangements did not show copy number changes. Copy number data for the tumor with a *EWSR1*::*NFATC2* fusion (**E**) were uninterpretable

RNA-seq was successfully performed on 10 of 12 cases; in 3 cases, the fusion transcripts were previously established. Pathogenic gene fusions involving *EWSR1* were present in 9 tumors, including 4 with *EWSR1*::*KLF15* fusion, 2 with *EWSR1*::*PBX3* fusion, 1 case each with *EWSR1*::*POU5F1* and *EWSR1*::*NFATC2* fusion (Fig. 2), and 1 case with *EWSR1* rearrangement by FISH but unsuccessful RNA-seq due to insufficient nucleic acid yield. Two additional cases had *IRF2BP2*::*CDX1* fusions. On hierarchical clustering, tumors containing shared fusions were relatively closely associated with each other (Supplementary file 3; Fig. S1).

Inferred relative copy number profiles of the epigenetically distinct MET group were evaluated from the DNA-MP data. Similar to the RNA-seq data, the copy number data also showed heterogeneity within this group. All four tumors with *EWSR1*::*KLF15* fusion showed recurrent loss of 3q and 22q. In contrast, no large-scale copy number changes were observed in other fusion-positive cases with interpretable copy number profiles (Fig. 2). Among the fusion-negative or fusion-unknown, cases 12 and 14 had distinct copy number gains and case 15 showed no copy number changes (Supplementary file 2/ Supplementary Table 1).

### EWSR1::KLF15 tumors (*n* = 4)

**Fig. 3:**
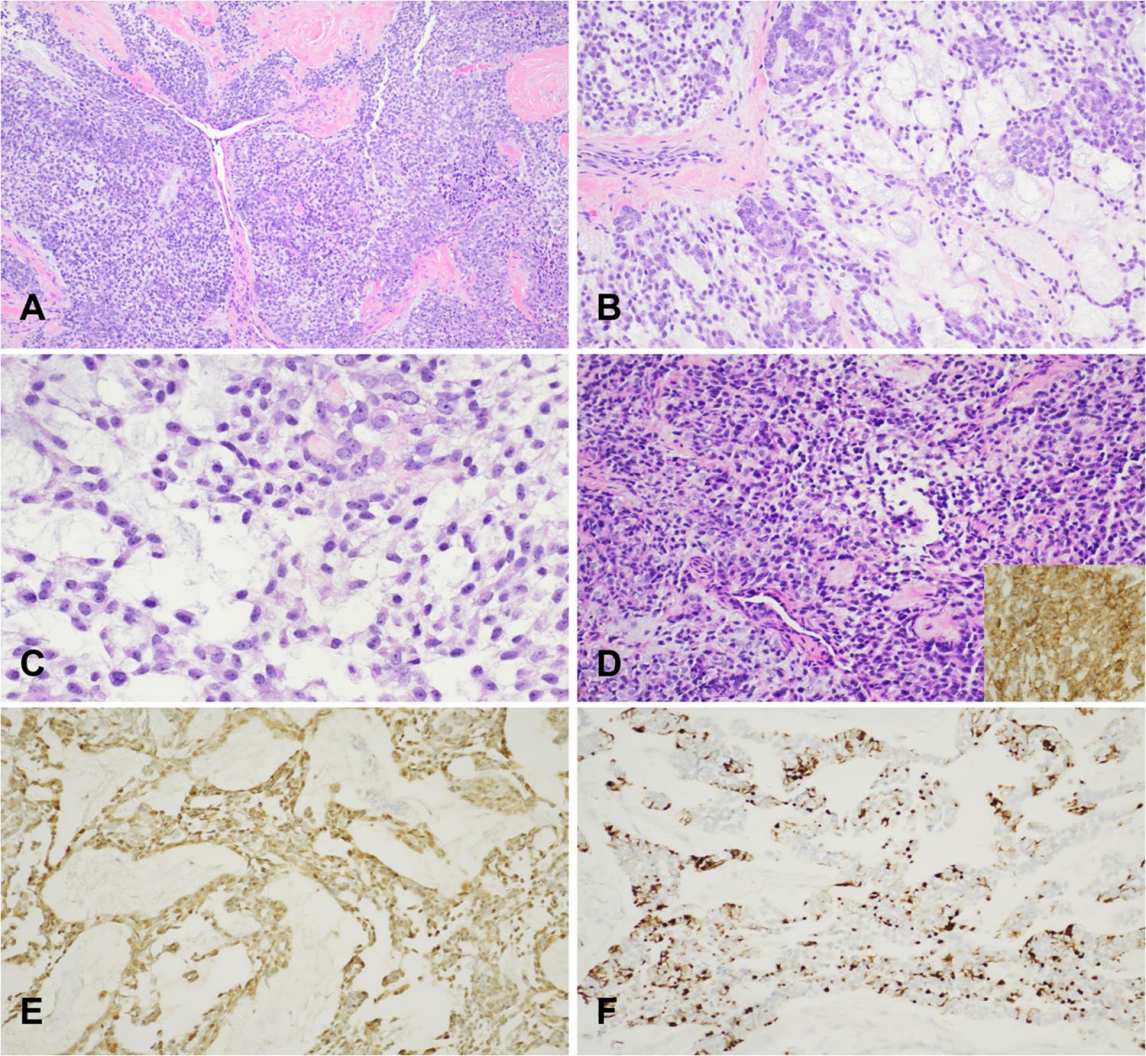
Histologic features of *EWSR1*::*KLF15*-rearranged myoepithelial tumors. **A**–**D** Tumors within this group showed sheets and aggregates of tumor cells in a fibromyxoid background with areas of reticular and nested arrangement. Nuclear atypia with prominent nucleoli were present in all cases. Primitive undifferentiated small round cell component was identified in 2 tumors (**D**) and showed CD99 expression (**D** inset). **E** Cytokeratin AE1/3 and **F** S100 staining in the tumor cells

Of the 15 METs that formed the epigenetically distinct group, 4 were closely associated on t-SNE dimensionality reduction (cases 1–4; Fig. 1B, Supplementary file 3; Fig. S1). All showed *EWSR1*::*KLF15* fusion identified by RNA-seq. Histologically, all four tumors showed overlapping architectural features characterized by radiating cords of spindle and epithelioid cells within a myxoid stroma (Fig. 3). Tumor cells displayed eosinophilic to clear cytoplasm. Nuclear atypia and nucleolar prominence were noted in all four cases. Both the primary and recurrent tumors (case 1 and 2) from the same patient displayed focal undifferentiated small cell populations. IHC identified CKAE1/3, EMA, and S100 positivity in all four tumors, SOX-10 in both tumors tested, SMA in three of four tumors, GFAP in two of four tumors, and retained SMARCB1 expression in all. Clinically, all tumors affected children at a median age of 2 years (range 2–3 years). The tumors involved soft tissue of the trunk and extremities of two patients and the face of one patient. Please refer to Supplementary file 2 for details of clinical and follow-up information.

### EWSR1::PBX3 tumors (*n* = 2)

**Fig. 4:**
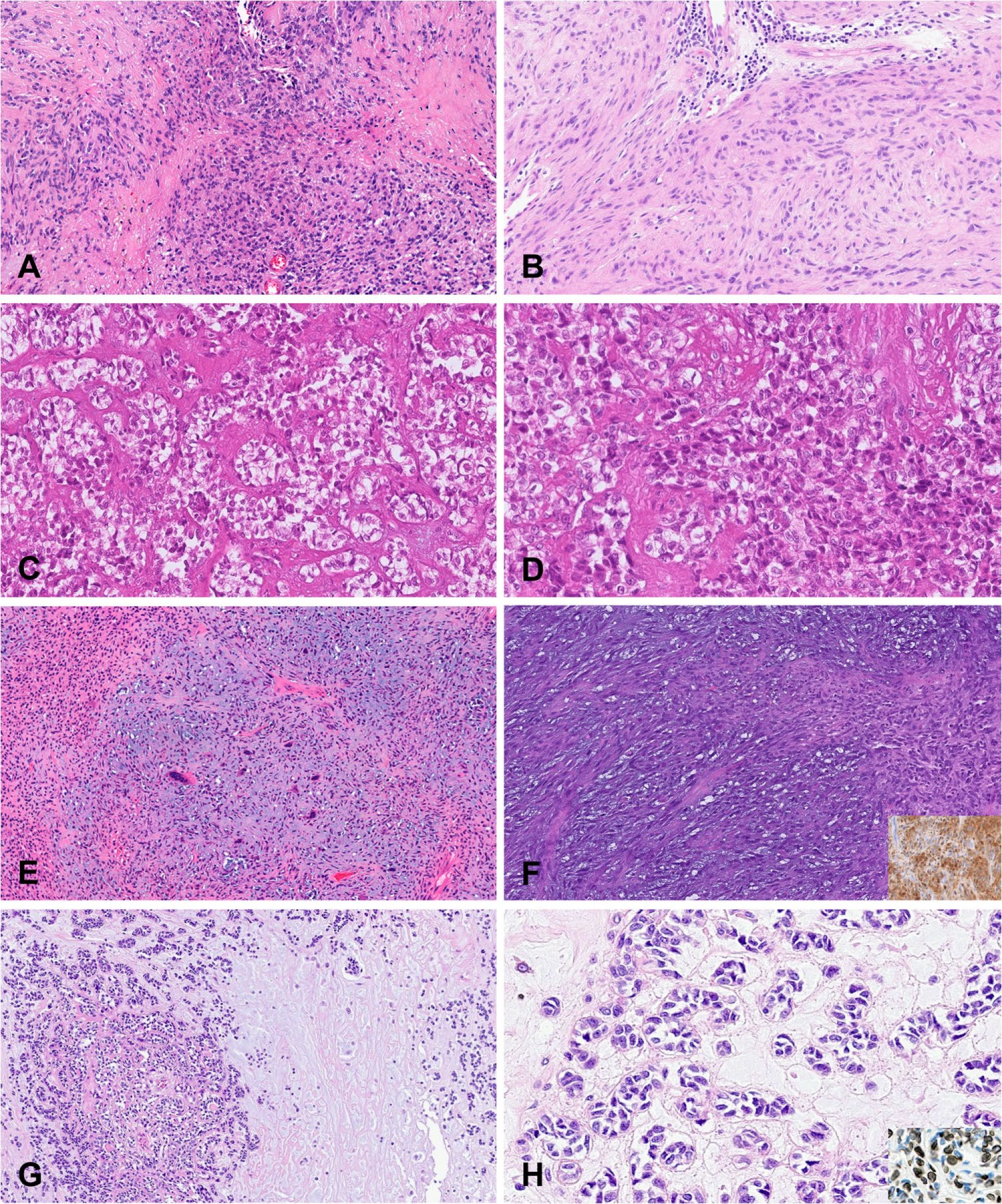
Histologic features of other fusion-positive myoepithelial tumors. **A**, **B** Both *EWSR1*::*PBX3*-rearranged tumors showed bland plump spindled and epithelioid tumor cells with a sclerotic background (**A**) and background small-caliber blood vessels showing perivascular lymphoid cuffing (**B**). Nuclear atypia was absent in both cases. The tumors expressed EMA (**A** inset) and S100 (**B** inset**)**. **C**, **D** The *EWSR1*::*POU5F1*-rearranged myoepithelial tumor showed nested arrangement of clear epithelioid tumor cells with areas showing nuclear atypia and prominent nucleoli (**D**). **E**, **F** Both *IRF2BP2*::*CDX1*-rearranged tumors arose in acral sites (hand and metatarsal region) and showed spindle cells within a myxochondroid stroma; one case also had interspersed osteoclastic giant cells (**E**). S100 shown in **F** inset. No chondrosarcomatous differentiation was present. **G**, **H** The *EWSR1::NFATC2*-rearranged neoplasm showed nested arrangement of epithelioid tumor cells within a myxohyaline stroma. The tumor cells expressed epithelial markers (CKAE1/3 shown in **H** inset)

Histologically, both *EWSR1*::*PBX3*-rearranged tumors (cases 5 and 6) showed nodular growth pattern, fascicles of spindle and epithelioid cells within collagenous stroma, and capillary-like blood vessels with associated lymphocytes (Fig. 4A, B), similar to previous descriptions [16, 17]. We found epithelial marker expression (EMA in both, CKAE1/3 in one) and S100 positivity in both tumors. GFAP and p63 were positive in 1 case and SMA was positive in both. Both tumors were SOX10 negative. Clinically, 1 tumor occurred in the soft tissue of the toe in a 50-year-old female (case 5); the other occurred in the left metatarsal soft tissue of a 31-year-old male (case 6). Follow-up information after surgical removal was unavailable (Supplementary file 2).

### EWSR1::POU5F1 tumor (*n* = 1)

The tumor with an *EWSR1*::*POU5F1* fusion (case 7) contained nested clear to eosinophilic tumor cells with EMA and S100 expression but was negative for other myoepithelial markers (Fig. 4C, D). Clinically, this tumor affected a 5-year-old female, arising in the deep soft tissue of the back. Follow-up information was unavailable (Supplementary file 2).

### IRF2BP2::CDX1 tumors (*n* = 2)

Histologically, both tumors with *IRF2BP2*::*CDX1* rearrangement (cases 8–9) showed a well-circumscribed lesion composed of bland epithelioid tumor cells in a chondromyxoid stroma (Fig. 4E, F). Both tumors showed thin-walled branching and focally ectatic vasculature in the background. We found scattered osteoclastic giant cells in 1 tumor (case 8). Cytokeratins and S100 were positive in both, while EMA was positive in one case (unavailable in the other); focal SMA expression was seen in one case. SOX10 was positive in the spindle cells in case 8. Clinically, both tumors affected acral sites. Please refer to Supplementary file 2 / Supplementary Tables for details of clinical and follow-up information.

### EWSR1::NFATC2 tumor (*n* = 1)

The *EWSR1*::*NFATC2* fusion (case 10) showed an encapsulated soft tissue neoplasm composed of clusters of epithelioid tumor cells forming acinar-like structures, within abundant myxoid stroma (Fig. 4G, H). The tumor cells co-expressed CKAE1/3, EMA, and S100, while SOX10, GFAP, p63, and SMA were negative. This tumor was spatially close to sclerosing epithelioid fibrosarcoma on t-SNE analysis. Clinically, this tumor arose in the soft tissue of the thigh in a 34-year-old male. Please refer to Supplementary file 2 for details of clinical and follow-up information.

### Fusion-negative/unknown tumors (*n* = 5)

**Fig. 5:**
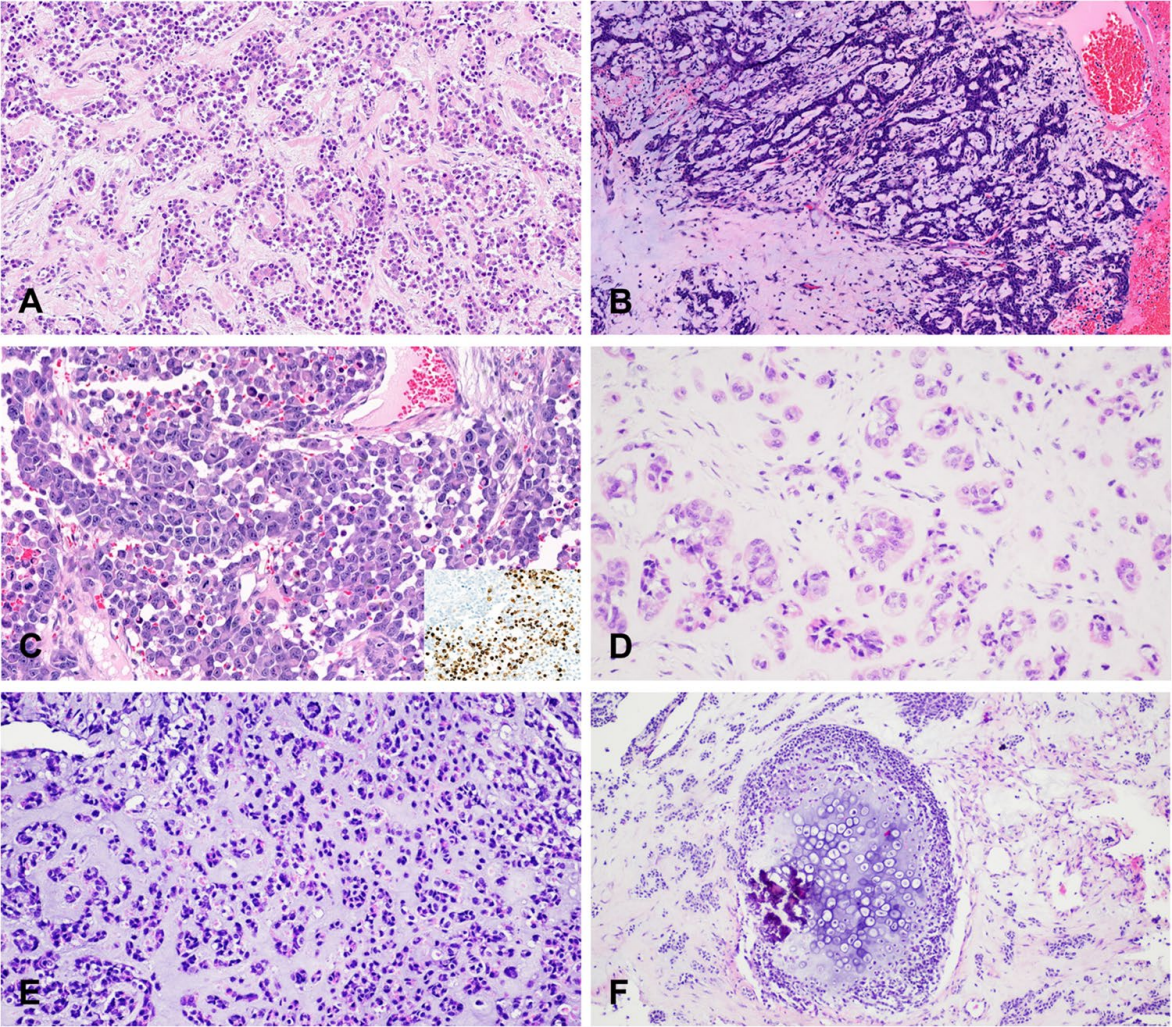
Histologic features of fusion-negative myoepithelial tumors. **A**, **B** Two myoepithelial tumors (cases 12 and 15) showed abundant fibromyxoid stroma in the background, with interconnecting nests and aggregates of epithelioid tumor cells. **C** Malignant myoepithelial tumor with rhabdoid and plasmacytoid features (case 13); CKAE1/3 expression is shown in inset. **D** Malignant myoepithelial tumor (case 11) with clusters of epithelioid tumor cells in a fibromyxoid background and S100 expression (**D** inset). **E**, **F** Malignant myoepithelial tumor (case 14) with clusters of epithelioid tumor cells and foci of hyaline cartilage nodules. GFAP positivity is shown (**F** inset)

The remaining five tumors in the epigenetic distinct group from the hierarchical cluster analysis (cases 11–15) did not form distinct clusters on t-SNE analysis and instead distributed individually. Histologically, four tumors showed fibromyxoid and hyalinized background, with one showing hyaline cartilage nodules (Fig. 5F). Tumor cells appeared in nests and aggregates. Myoepithelial markers were variably expressed: all five cases showed epithelial marker expression, with co-expression of S100 (four of five), SOX10 (four of five), GFAP (three of five), p63 (two of five), and/or SMA (three of five). Because case 13 grouped near cutaneous melanoma by t-SNE analysis, we evaluated multiple melanocytic markers (HMB-45, Melan-A, MiTF) that were all negative. The tumors showed variable clinical features and presentation. The tumors presented at a median age of 33 years (range, 8–61 years) in the extremities (three of five) and head and neck (two of five). Details are listed in Supplementary file 2.

## Discussion

In this study, we used a combination of histopathologic and molecular approaches to characterize tumors institutionally diagnosed as METs. Our analysis supports that at least a subset of METs is epigenetically distinct from other soft tissue tumors but METs still are characterized by significant underlying genomic diversity. Specifically, we find an epigenetically distinct group on hierarchical analysis and looser association of small groups by t-SNE, suggesting METs could represent a broad tumor family encompassing several molecular subtypes. Our analysis also validates the importance of applying strict histomorphologic and immunophenotypic criteria in the diagnosis of METs, as we find it holds high sensitivity, albeit low specificity, for molecularly defined tumors.

The epigenetically distinct MET group was enriched for driver fusions, especially involving *EWSR1*. Most of these drivers are well-documented in METs [3, 9, 18].

Histologically, the fusion-positive tumors aligned with previous clinical and morphologic descriptions [9, 16, 19]. For example, the *EWSR1*::*KLF15*-fused tumors involved pediatric patients, showed characteristic morphology of corded aggregates of tumor cells within a myxohyaline background and had undifferentiated round cells in a subset [9, 20, 21]. These tumors formed a small but well-defined group on t-SNE analysis and contained similar copy number profiles. Tumors with *EWSR1*::*POU5F1* or *EWSR1*::*PBX3* fusions also showed histology aligning with previous descriptions [3, 20]. We also observed that the primary and relapsed tumor in the same patient maintained the core methylation signature.

We identified three tumors in our epigenetically distinct METs group that carried fusions described in molecularly defined, non-MET tumor types, i.e., *EWSR1*::*NFATC2*-rearranged mesenchymal neoplasms and *IRF2BP2*::*CDX1*-rearranged neoplasm [21, 22]. Early descriptions of *EWSR1*::*NFATC2*-rearranged mesenchymal neoplasms placed them in the category of Ewing-like undifferentiated round cell sarcoma. However, there is increasing evidence that these are distinct from conventional Ewing sarcoma based on their clinical, biologic, transcriptional, and DNA methylation characteristics [23, 24]. The relationship of *EWSR1*::*NFATC2* tumors to METs, despite their “imperfect” myoepithelial immunophenotype, has been described by others [25]. Notably, our case shows MET immunophenotype. Similarly, *IRF2BP2*::*CDX1* fusion has been recently described in rare mesenchymal chondrosarcoma cases [21, 22]; Patton et al. recently reported a variant fusion in an intravascular myoepithelioma [26]. Our findings suggest that tumors with these fusions may be epigenetically similar to the broad family of METs. Future studies with better representation of such tumors are needed to understand the relationship between these histologically and molecularly overlapping entities, as our cohort exhibited significant molecular diversity. The distinction is clinically relevant, as there are treatment differences across these tumor types.

In addition to identifying an epigenetically distinct group, we found that a proportion of tumors, institutionally diagnosed as METs because of the consistent histology and immunophenotype, showed epigenetic features suggesting an alternative diagnosis. A subset of these carried the hallmark molecular abnormalities of the suggested entities. Notably, the rate of misclassification could be substantially reduced by applying strict morphologic and immunophenotypic criteria — in our series, the overall misclassification rate for institutionally diagnosed METs was 50% (15/30), but with application of the strict criteria, the misclassification rate for histologic “consistent” METs dropped to 32% (7/22). Despite this reduction, we still identified cases that fit the histologic criteria and could ultimately be reclassified as another tumor. This observation suggests that addition of METs to supervised methylation-based classification models may be useful especially for challenging or ambiguous cases, or where the RNA quality/quantity is insufficient for fusion testing.

Recent studies demonstrate that some molecularly defined entities have the potential to resemble myoepithelioma [25, 27–29]. The accurate classification of these “myoepithelioma-like” tumors hinges on the identification of underlying fusions. In our cohort, these misdiagnoses represented variants of tumors showing ambiguous epithelioid or spindle cell histology and aberrantly expressing myoepithelial markers. Our cases include underrecognized mimickers, including EMCS expressing epithelial markers and biphasic synovial sarcoma with hyalinizing and sclerosing histology and predilection for the distal lower extremity [25, 29, 30].

Our findings demonstrate that co-expression of cytokeratin and/or EMA with S100 or SOX10 or GFAP, in the correct morphologic context, is generally supportive of METs. All tumors that fell in the epigenetic distinct MET group also met the recommended histomorphologic criteria, and tumors that did *not* meet the strict MET criteria fell outside the distinct epigenetic group. The classic phenotype may not be present in all METs [1, 31], and therefore, diagnosis requires careful interpretation of histology, clinical data, and molecular information.

We also observe that a subset of fusion-negative tumors with myoepithelial features and SMARCB1 loss is epigenetically related to ES and MRT and are  distinct from fusion-positive and other fusion-negative METs. SMARCB1 loss is known to occur in a subset of myoepithelial carcinomas lacking gene fusions [32, 33]. Whether this subset represents ES or MRT with MET features, or a histologically distinct MET-like tumor with SMARCB1 loss is unclear currently and requires further investigation.

While our study offers evidence for an epigenetically distinct MET group, the cohort is relatively small. Nonetheless, it lays groundwork for additional studies to validate our findings. Those studies are needed to fully define the molecular diversity within METs and to understand the drivers of morphologic and clinical heterogeneity.

In conclusion, our findings support the concept that METs are an epigenetically diverse group of neoplasms with some unifying histologic and immunophenotypic features, with various molecular drivers. We show that DNA-MP is a potentially useful orthogonal method to distinguish morphologic mimics with “myoepithelioma-like” features. We stress the importance of interpreting the *specific* myoepithelial immunophenotype in the correct morphologic context and with molecular diagnostic input, especially in the setting of the described pitfalls.

## Supplementary Information

Below is the link to the electronic supplementary material.

Supplementary file1 (DOCX 36 KB)

Supplementary file 2/ supplementary Table (XLSX 18 KB)

Supplementary file3 (DOCX 13416 KB)

## Data Availability

Raw DNA methylation data generated for this study was deposited in Gene Expression Omnibus (GEO) under the accession GSE243075.
