# Supplementary material for: Reappraisal of soft tissue myoepithelial tumors by DNA methylation profiling reveals an epigenetically distinct group of mostly fusion-driven neoplasms
Source: Virchows Arch. 2024 Dec 5;486(3):573–84. doi: 10.1007/s00428-024-03977-4 (PMC11949712; doi:10.1007/s00428-024-03977-4)
Supplement: Supplementary file 1 — Supplementary file1 (DOCX 36 KB) [file 428_2024_3977_MOESM1_ESM.docx]

**SUPPLEMENTARY METHODS**

**DNA methylation profiling**

Methylation analysis was performed using the Illumina Infinium Human MethylationEPIC BeadChip (850K) on 250-500 ng of DNA extracted from formalin-fixed paraffin-embedded (FFPE) tissue. Methylation data were preprocessed using the *minfi* package (v.1.28.3) [1] in R (<http://www.r-project.org>, version 3.5.1). Functional normalization [2] with NOOB background correction and dye-bias normalization [3] was performed. The preprocessing step also included calculation of beta values and detection *p* values. All samples had detection *p* values less than 0.005. Probes located on sex chromosomes, containing nucleotide polymorphisms (dbSNP132 Common) within 5 base pairs of and including the targeted CpG site, mapping to multiple sites on hg19 (allowing for 1 mismatch), or showing cross-reactivity were removed from analysis. After filtering, there were 408,864 methylation loci remaining.

**Unsupervised analysis**

Unsupervised analysis of methylation data was performed by hierarchical cluster analysis and t-distributed stochastic neighbor embedding (t-SNE). The hierarchical cluster analysis was performed and visualized using the R *circlize* (v 0.4.16) package.  The pairwise distances were measured by taking the inverts of the Spearman correlations [4] between all pairs of the 348 MET and the reference sarcoma samples. The hierarchical clustering was performed on these distances using the Ward’s minimum variance method (specifically, Ward’s D2 criterion) [5]. The t-distributed stochastic neighbor embedding (t-SNE) was performed using *Rtsne* (v.0.17). Briefly, principal components were calculated using the 5,000 most variably methylated probes as measured by the standard deviation of the probe-level beta values across samples. The same probes were used for principal component analysis. The number of statistically significant principal components was computed using the agDimension function in the *PCDimension* package (v.1.1.11). Principal component scores for all statistically significant components (*k* = 15) were used for t-SNE analysis [6]. The following non-default parameters were used: theta = 0.5, pca = F, perplexity = 50. This approach was used for the combined analysis of sarcoma reference samples GSE140686 [7] and the 30 MET cases.

**Copy number variant analysis**

Copy number profiles were generated from genome-wide DNA methylation data using the *conumee* package (v1.36.0) in R [8] as previously described [9], using the threshold values of 0.18 for copy number gain and -0.2 for copy number loss.

**Molecular fusion analysis**

RNA sequencing (RNA-seq) was attempted on all tumors with sufficient material. RNA was extracted from FFPE tumor tissue by using Maxwell® CSC FFPE RNA Extraction Kit (Promega, Madison, WI, USA) for automatic extraction. Total RNA (300 ng) was used for next-generation sequencing library preparation with SureSelect XT HS2 kit (Agilent Technologies, Santa Clara, CA, USA) following the manufacturer's instructions. Libraries were pooled and the sequencing run was performed in paired-end mode (2 × 101 bp reads) using the NextSeQ 550 system (Illumina, San Diego, California) to generate at least 20 million reads per sample. Raw reads were preprocessed using Fastp to remove low-quality reads and any adapters and were then aligned to the reference human genome (UCSC-Build38) by using the STAR (2.5.3a) algorithm [10]. The resulting alignment files were used to identify any candidate fusion transcripts by using Arriba and FusionCatcher pipelines [11, 12]. Gene counts were obtained using Cufflinks (v2.2.1) and differentially expressed genes were identified by DEseq2 (version 3.3).

In one case, fluorescence *in situ* hybridization (FISH) was performed on representative FFPE sections from the tumor by using a commercially available capicua transcriptional repressor (*CIC*) (19q13.2) dual-color [3′ telomeric (SpecOrange)/5′ centromeric (SpecGreen)], break-apart probe (Empire Genomics; catalog number CIC-BA). All molecular results were reviewed by an experienced board-certified molecular pathologist (S.C.K.).

**Immunohistochemistry for case re-evaluation**

Immunohistochemistry (IHC) was performed on selected cases using the following antibodies: H3K27me3 (clone C36B11, 1:200, Cell Signaling Technology), WT-1 [clone 6F-H2, ready to use (RTU), Cell Marque] and DUX4 (clone P4H2, 1:200, Thermo Fisher), and HMB-45 (clone HMB45, 1:20, Dako), Melan-A (PA0044, RTU, Leica Microsystems), MiTF (M3621; 1:200; Dako). The results of these IHCs were reviewed by two pathologists (FM and SCK).

**SUPPLEMENTARY RESULTS**

**Clinicopathologic and molecular findings of soft tissue tumors with myoepithelial features reclassified to reference entities by DNA methylation profiling**

We analyzed the 15 tumors that were reclassified as other entities by unsupervised analysis. Of these, 7 of 15 tumors were histologically in the “Consistent” group. All 7 tumors showed mostly epithelioid and spindled cytologic features with varying amounts of stroma (Supplementary Fig. 2–3). All tumors expressed myoepithelial markers, including a combination of epithelial markers (CKAE1/3 in 3, EMA in 7) with S100 (7 cases), SOX10 (2 cases), GFAP (1 case) SMA (4 cases), or p63 (1 case). RNA-seq was successfully performed on 6 of 7 cases. For cases 16 and 17, which grouped respectively with extraskeletal myxoid chondrosarcoma (EMCS) and synovial sarcoma, the DNA-MP prediction was validated by RNA-seq. The molecularly confirmed EMCS showed EMA and S100 expression (Supplementary Fig. S2A–D). The molecularly confirmed synovial sarcoma showed predominantly epithelioid histology with focal S100 expression, serving as pitfalls (Supplementary Fig. S2E–H). For the remaining 5 cases (Supplementary Fig. S3), tumor type–defining molecular abnormalities do not exist (3 of 5) or could not be identified in our cohort (2 of 5). Therefore, these remaining 5 cases were considered unresolved between MET and the predicted tumor type.

The remaining 8 of 15 reclassified tumors by DNA-MP represented institutionally diagnosed METs that were in the histologically “Questionable” group (Supplementary Fig. S4). RNA-seq was successful in 5 cases. Of these, 4 were negative for fusions while 1 showed a *PHF1*::*TFE3*-rearrangement, confirming the DNA-MP predicted OFMT classification. Additional IHCs were performed in 2 cases. In case 25, the DNA-MP suggested a diagnosis of CIC-rearranged undifferentiated round cell sarcoma. The tumor failed RNA-seq and FISH analysis for *CIC* breakapart probe (possibly due to archival material). In this case, CD99, WT-1, and DUX4 (performed to validate the DNA-MP prediction) were positive, supporting the predicted diagnosis. In case 29, where the suggested diagnosis was MPNST, the H3K27me3 immunostain failed to show internal control positivity repeatedly.

**SUPPLEMENTARY REFERENCES**

1. Aryee MJ, Jaffe AE, Corrada-Bravo H, et al. (2014) Minfi: a flexible and comprehensive Bioconductor package for the analysis of Infinium DNA methylation microarrays Bioinformatics 30:1363-1369. doi: 10.1093/bioinformatics/btu049

2. Fortin JP, Labbe A, Lemire M, et al. (2014) Functional normalization of 450k methylation array data improves replication in large cancer studies Genome Biol 15:503. doi: 10.1186/s13059-014-0503-2

3. Triche TJ, Jr., Weisenberger DJ, Van Den Berg D, et al. (2013) Low-level processing of Illumina Infinium DNA Methylation BeadArrays Nucleic Acids Res 41:e90. doi: 10.1093/nar/gkt090

4. Spearman C (1904) The proof and measurement of association between two things The American Journal of Psychology 15:72-101. doi: 10.2307/1412159

5. Ward Jr JH (1963) Hierarchical grouping to optimize an objective function Journal of the American statistical association 58:236-244

6. Van der Maaten L, Hinton G (2008) Visualizing data using t-SNE Journal of machine learning research 9

7. Koelsche C, Schrimpf D, Stichel D, et al. (2021) Sarcoma classification by DNA methylation profiling Nat Commun 12:498. doi: 10.1038/s41467-020-20603-4

8. Hovestadt V, Zapatka M, Hovestadt MV, et al. (2016) Package ‘conumee’

9. Clay MR, Pinto EM, Cline C, et al. (2019) DNA Methylation Profiling Reveals Prognostically Significant Groups in Pediatric Adrenocortical Tumors: A Report From the International Pediatric Adrenocortical Tumor Registry JCO Precision Oncology:1-21. doi: 10.1200/po.19.00163

10. Dobin A, Davis CA, Schlesinger F, et al. (2013) STAR: ultrafast universal RNA-seq aligner Bioinformatics 29:15-21. doi: 10.1093/bioinformatics/bts635

11. Uhrig S, Ellermann J, Walther T, et al. (2021) Accurate and efficient detection of gene fusions from RNA sequencing data Genome Res 31:448-460. doi: 10.1101/gr.257246.119

12. Nicorici D, Şatalan M, Edgren H, et al. (2014) FusionCatcher–a tool for finding somatic fusion genes in paired-end RNA-sequencing data biorxiv:011650
