# Supplementary material for: Reappraisal of soft tissue myoepithelial tumors by DNA methylation profiling reveals an epigenetically distinct group of mostly fusion-driven neoplasms
Source: Virchows Arch. 2024 Dec 5;486(3):573–84. doi: 10.1007/s00428-024-03977-4 (PMC11949712; doi:10.1007/s00428-024-03977-4)

**Figure S1.** Unsupervised analysis of institutionally classified myoepithelial tumor (MET) samples (as shown in Figure 1 in main document) with annotation of fusion-positive MET cases. [Color key includes the reference tumor entities^11^ included in the unsupervised analysis; red dots and bars denote the MET study cohort. Abbreviations: ASPS, alveolar soft part sarcoma; CB, chondroblastoma; CSA, chondrosarcoma; CSA-CC, clear cell chondrosarcoma; CSA-IDH, chondrosarcoma (IDH mutant); CTRL-MUS, skeletal muscle control; EMCS, extraskeletal myxoid chondrosarcoma; ES, epithelioid sarcoma; ESS-HG, high-grade endometrial stromal sarcoma; FDY, fibrous dysplasia; MEL-CUT, cutaneous melanoma; MPNST, malignant peripheral nerve sheath tumor; MRT, malignant rhabdoid tumor; OB, osteoblastoma; OFMT, ossifying fibromyxoid tumor; SBRCT-CIC, capicua transcriptional repressor (*CIC*)-rearranged small blue round cell tumor; SCC-CUT, cutaneous squamous cell carcinoma; SEF, sclerosing epithelioid fibrosarcoma; SYSA, synovial sarcoma; USARC, undifferentiated sarcoma].

**Figure S2.** Histologic features of tumors with myoepithelial features predicted as reference tumors by DNA methylation profiling and validated by RNA sequencing. **(A–C)** Extraskeletal myxoid chondrosarcoma (EMCS) presented in the abdominal wall of a 35-year-old male (case 21) with characteristic histology **(A)** and areas resembling myoepithelial tumors **(B–C)**, with EMA positivity **(D)**. By unsupervised hierarchical clustering, the tumor classified as EMCS; RNA-seq identified *TAF15*::*NR4A3* fusion. **(D–F)** Synovial sarcoma with myoepithelioma-like features presenting in the ankle of a 35-year-old female (case 22). The tumor showed predominantly epithelioid features in a hyalinized stroma **(E–G)** with rare foci of typical spindle cell features **(E)**, and focal S100 expression **(H)**. By unsupervised hierarchical clustering, the tumor classified as synovial sarcoma; RNA-seq identified *SS18*::*SSX2* fusion.

**Figure S3.** Histologic features of tumors with canonical myoepithelial features predicted as reference tumors by DNA methylation profiling. **(A–B)** Myxoid-rich neoplasm of the vulvar region of a young female, histologically classified as a myoepithelial tumor (case 16). This tumor was histologically characterized by epithelioid and plump spindled tumor cells with nuclear clearing, and prominent nucleoli, within a myxoid background. This tumor expressed EMA, S100, and ER and showed loss of SMARCB1 expression **(B inset)**. DNA methylation predicted a diagnosis of epithelioid sarcoma in this case **(C–D)**. Epithelioid tumor with myxoid and hyalinized stroma and myoepithelial immunophenotype (case 17). DNA methylation predicted a diagnosis of epithelioid sarcoma in this case**. (E)** This tumor in an infant male showed a malignant epithelioid neoplasm with a myxoid background, expressing myoepithelial markers, with loss of SMARCB1 (case 20). DNA methylation predicted a diagnosis of malignant rhabdoid tumor. **(F)** This tumor from the inguinal region of a 49-year-old was classified as a myoepithelial tumor based on the histologic features and immunophenotype. DNA methylation predicted a diagnosis of extraskeletal myxoid chondrosarcoma (EMCS)

**Figure S4.** Tumors with myoepithelial morphology but incomplete immunophenotype. **(A–C)** *CIC*-rearranged undifferentiated round cell sarcoma showing epithelioid tumor cells in cords and aggregates in a myxohyaline-rich background (case 25). The tumor cells showed diffuse SMA expression (reason for misclassification). Molecular assays failed to detect the underlying alteration. However, there was diffuse nuclear positivity for DUX4 **(C)**, WT-1, and CD99 expression (not shown), supporting the prediction. **(D–F)** Ossifying fibromyxoid tumor with hyalinized stroma and lack of characteristic shell of bone (case 26). The tumor cells showed nodular arrangement **(D)** with epithelioid appearance and cytoplasmic clearing **(E)** and expression of CKAE1/3 **(F)**. SMARCB1 was lost within the tumor in a mosaic pattern (not shown). The tumor had a *PHF1*::*TFE3* fusion by RNA-seq. **(G–H)** Tumors classified as myoepithelial neoplasms but reclassified as other tumor types by DNA methylation. **(I)** Case 30 showed myoepithelial immunophenotype but absence of characteristic histology.


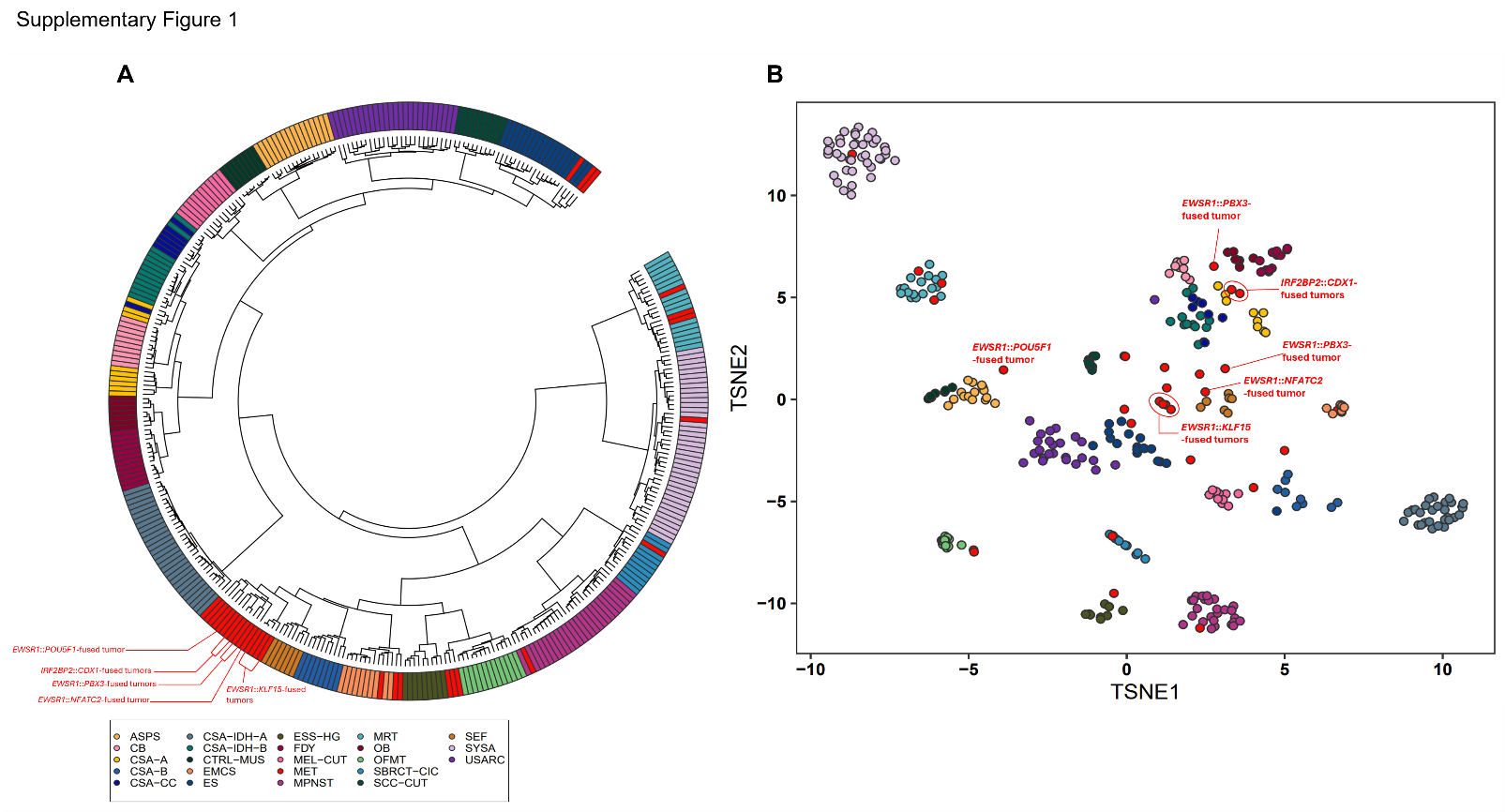

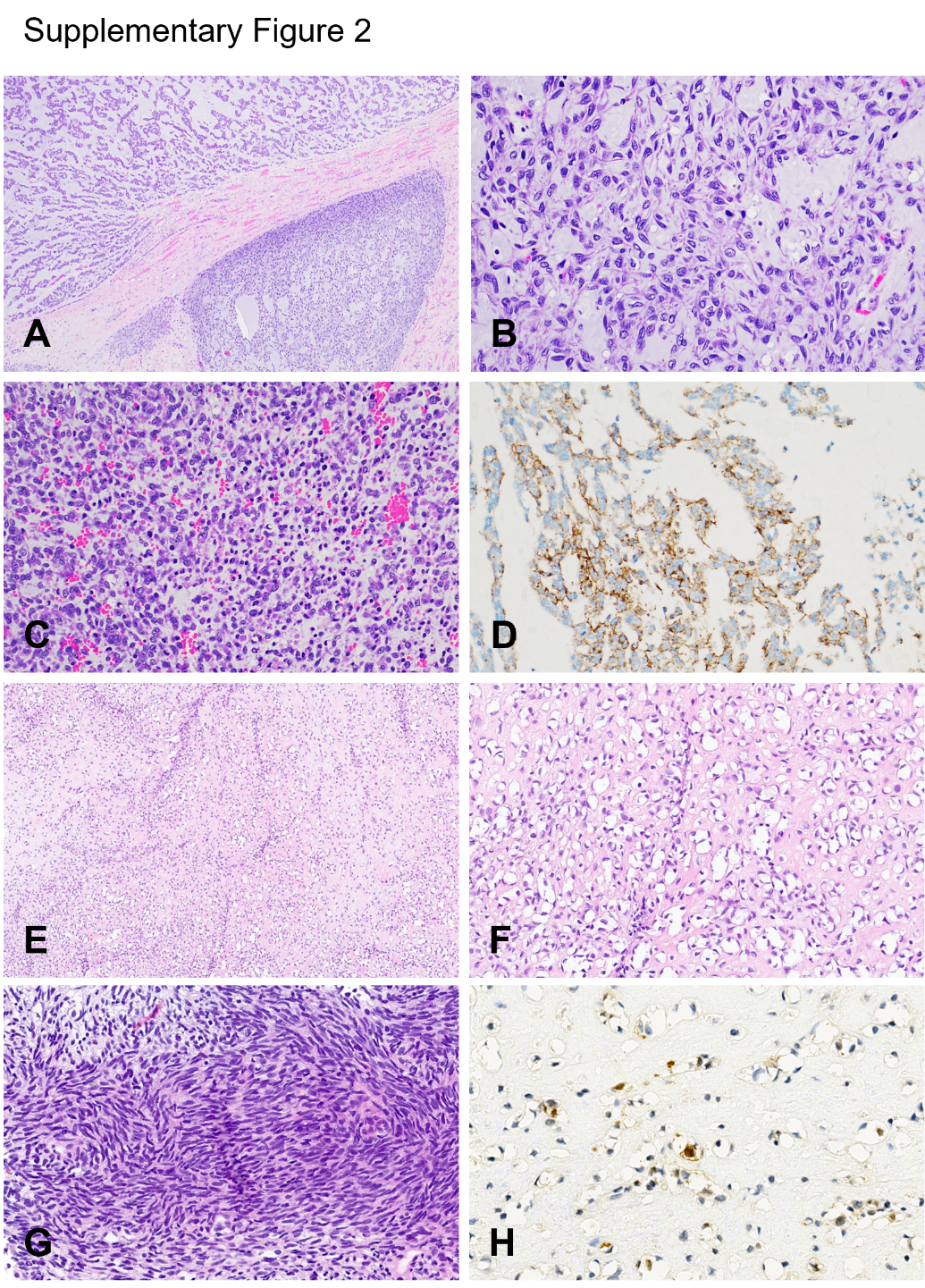

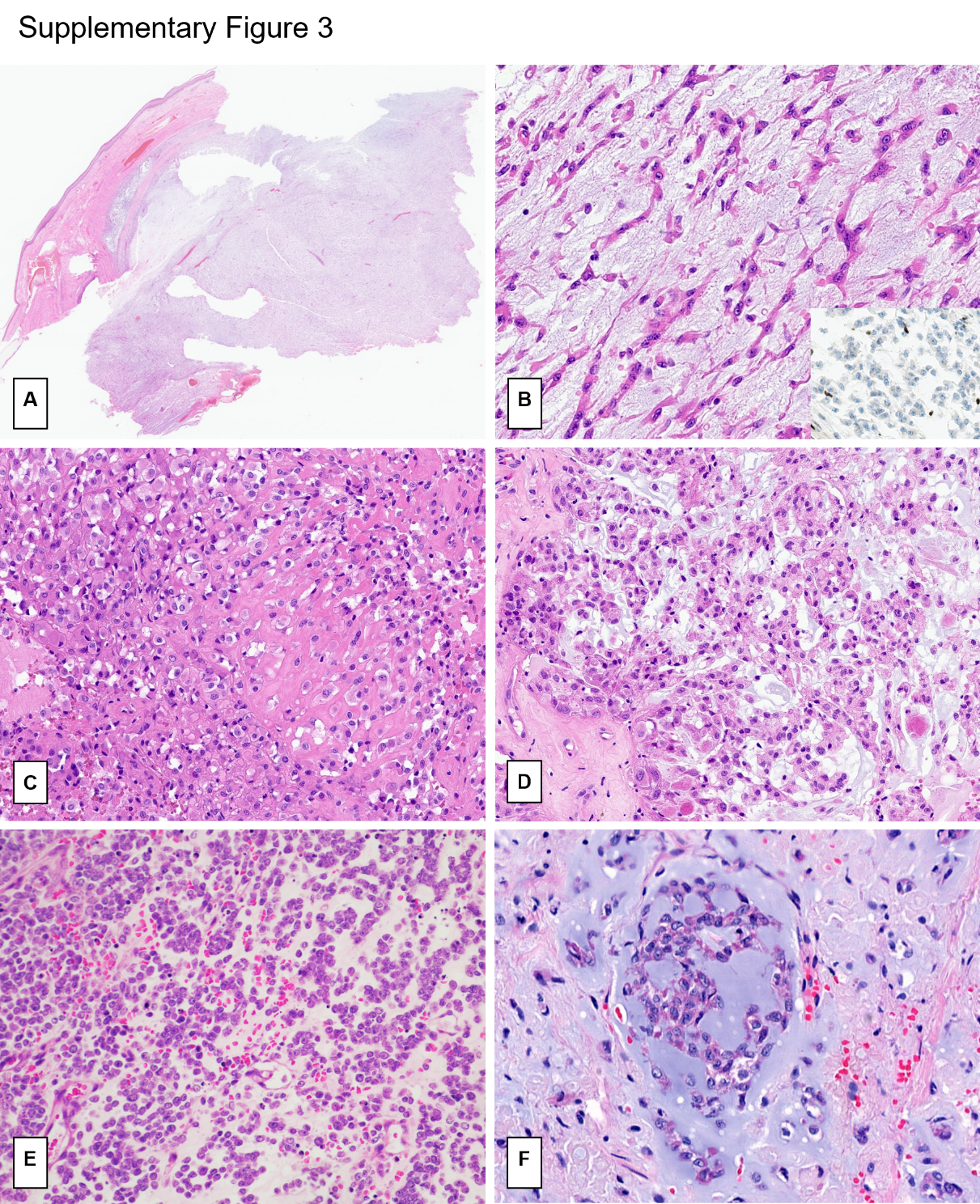

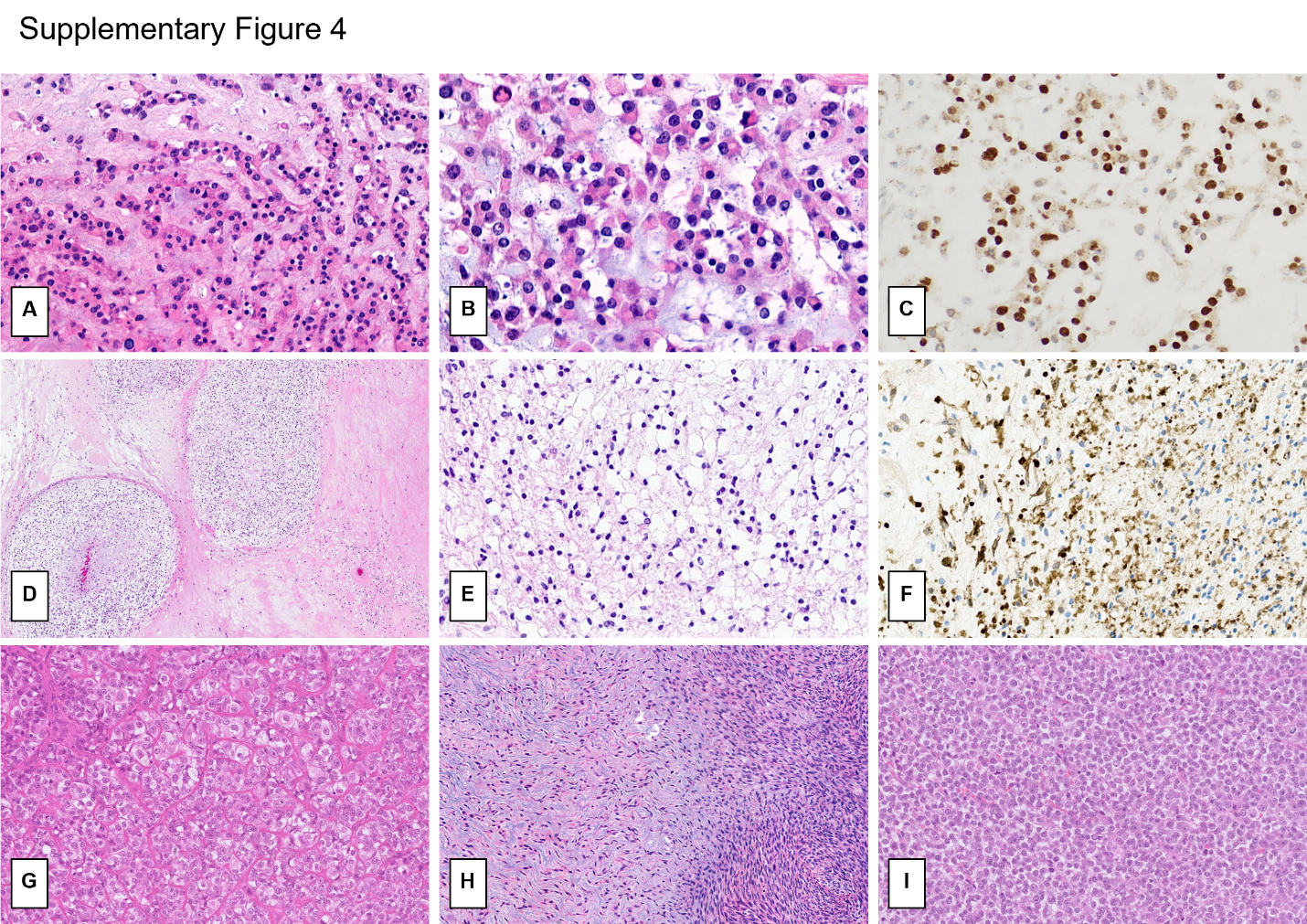

Supplement: Supplementary file 3 — Supplementary file3 (DOCX 13416 KB) [file 428_2024_3977_MOESM3_ESM.docx]
